# Investigation of a New Screw Press Forming Process for Manufacturing Connectors from ZK60 Magnesium Alloy Preforms

**DOI:** 10.3390/ma16093467

**Published:** 2023-04-29

**Authors:** Anna Dziubinska

**Affiliations:** Metal Forming and Casting Department, Faculty of Mechanical and Industrial Engineering, Warsaw University of Technology, Pl. Politechniki 1, 00-661 Warsaw, Poland; anna.dziubinska@pw.edu.pl

**Keywords:** metal forming, screw press, die forging, connector, forgings, cast preforms, ZK60 magnesium alloy

## Abstract

This article discusses a new technology of forming connector forgings from ZK60 magnesium alloy preforms by die forging on a screw press. The purpose of the study was to evaluate the feasibility of using preforms cast from the ZK60 magnesium alloy to forge a connector forging with improved mechanical properties compared to those obtained by casting. It also aimed to establish whether this new forging method has the potential for replacing the multi-stage forging process conducted on hydraulic presses used for high-strength Mg alloys. A numerical analysis of the proposed approach was performed by the finite element method, applying the popular DEFORM computer software for simulating forming processes. The numerical results confirmed that the developed method produces parts with the desired shape. The numerical results also provided information regarding the behavior of the workpiece’s material and the screw press forging process, including the distributions of strains and temperatures, the Cockcroft–Latham damage criterion, and energy required to form connector forgings. The proposed screw press forging process for producing ZK60 alloy connectors from cast preforms was verified by experimental tests. The connector forgings produced from the ZK60 magnesium alloy were then subjected to qualitative tests.

## 1. Introduction

The use of lightweight metals as construction materials is regarded to be of crucial importance for the future of various industries. Since the global trend is to use high-strength materials, there is a growing interest in lightweight magnesium metal alloys [1,2,3]. The application of these alloys in the automotive and aviation industries enables a significant weight reduction, thereby leading to lower fuel consumption and emissions as well as higher operating parameters of vehicles and aircrafts [4,5,6]. Modern technologies for producing parts made of magnesium alloys are constantly improved, which opens up new opportunities for their use in the automotive and aviation sectors [7,8,9]. In view of the foregoing, it is worth focusing on new metal-forming technologies for magnesium alloys, as they allow parts with higher strength and better functional properties to be produced when compared with those produced by casting. In the forging of magnesium alloys, it is necessary to ensure correct process variables, in particular the strain rate and temperature, in order to obtain suitable workability of the material. There are few industrial plants in the world that specialize in forging magnesium alloys. The forging of such alloys is usually conducted with hydraulic presses [10,11]. To make sure that forging process conditions are as close as possible to isothermal conditions, the tools are fitted with specially designed heating systems [10]. Stringent requirements for machines and devices pose a serious limitation for many forging plants. In light of the above, the author of this study has undertaken research to design a new forging method for magnesium alloys that uses screw presses—i.e., forging machines allowing for a dynamic deformation of the workpiece. Screw presses utilize kinetic energy accumulated in a flywheel coupled with a non-self-locking screw, which when rotating in a nut, translates rotational motion into forward progression transferred to the press’ slide [12,13]. This energy is then translated into deformation and pressure. A significant characteristic of screw presses is that they possess no kinematic limits for the progression of the slide. This means that the forging process may be executed until die faces meet. As a consequence, the dimensions of forgings in their height are the most precise among the available forging methods. In the event when the blow energy of the die is insufficient for the two die faces to meet, another blow is applied. Such an approach eliminates incomplete forging. Simulation methods are used in the analysis of existing technologies in the industry and in the design of new technologies for the production of forgings on the screw press. Numerical simulations of metal forming processes on a screw press provide an opportunity to analyze and optimize these, eliminating the need for costly industrial tests. Multivariate analysis enables optimal process parameters to be established, the number of operations and tool shape to be determined, and final properties of products to be predicted. The modeling of plastic forming processes requires the knowledge of physical quantities and phenomena that characterize not only the material being deformed but the entire system as well. For the numerical analysis, the finite element method (FEM) and the finite volume method (FVM) are used. Computer simulations of metal forming processes are based on the finite element method—an advanced method of solving systems of differential equations, based on the division of an area (continuous medium) into a finite number of elements (so-called discretization), for which the solution is approximated by specific functions and actual calculations are carried out only for the nodes of this division. The following FEM software is used to simulate forming processes on a screw press: DEFORM 3D; Forge; MSC.SuperForm; Qform; Simufact.Forming [4,14,15,16].

The finite volume method is based on Euler’s solution—which is what makes a difference in relation to the FEM. In the FEM, the mesh of elements generated in the material being deformed undergoes continuous changes in geometry, reflecting changes in the shape of the material being formed. In contrast, Euler’s solution involves generating a mesh of cubic elements (finite volumes) in the space where the deformed material is located. This mesh is rigidly fixed in space, and its parameters do not change during the calculation cycle. The following FVM software is used to simulate forming processes on a screw press: MSC.Dytran; MSC.SuperForge; Simufact Forming [17,18,19]. The development of a forging method that does not require the use of expensive tooling would significantly increase the number of forging plants manufacturing forged components made of magnesium alloys, and at the same time, would help reduce manufacturing costs.

This article presents the numerical and experimental results of a new screw press forging process for manufacturing connectors from ZK60 magnesium alloy preforms. The ZK60 magnesium alloy, which is in the Mg-Zn-Zr alloy group, was selected for the study [5,20]. The ZK60 alloy, which contains 6% magnesium, 0.45% zinc and zirconium, is one of the well-known wrought alloys. It is used to produce high-strength automotive and aviation parts [5,9,21]. It is characterized by slightly better forging applicability than other magnesium alloys [11]. In addition, due to its practical use, this alloy has been thoroughly investigated by different authors [22,23,24,25].

Currently, automotive and aviation connectors made from magnesium alloys are formed from casts by machining [26,27], casting, or multistage forging on hydraulic presses equipped with expensive installations for heating the tools. The manufacture of magnesium alloy connectors uses the machining technology, which has been discussed in the literature [28]. The machining of connectors consists in creating the required shape, dimensions, and quality of the surface by removing material from the cylinder or cuboid charge using cutting tools. This technique is very labor-intensive, time-consuming, energy-intensive and generates significant material losses and low quality of the formed products.

The manufacturing of the connectors from magnesium alloys by the casting technology offers products that have much lower mechanical and functional properties than parts obtained by metal forming methods, as presented in the literature [29,30]. Cast connectors have defects in casting such as structural heterogeneity, coarse grain size, blisters, porosity, shrinkage cavities, and microshrinkage, which result in their degraded properties. Forming processes described in the literature [31] provide the best strength properties for connectors used in the automotive industry. An example is die forging, as described in the specialized literature [32,33]. The multistage forging approach for the manufacturing of connector forgings uses wrought material in the form of a cylindrical billet (rod with a volume of 123,094 mm^3^) and consists of three operations: bending, preforming, and finishing. The process is conducted with a significant flash allowance. About 50% of the weight of the forging is process waste for flash. Additionally, the process uses expensive tool-heating systems. The use of the cast preform in the new screw press forging process would enable the component to be formed in a single operation and would improve the component’s properties compared with products obtained from machined castings. However, obtaining specific mechanical properties requires a certain level of strain within the forged part to be induced. Therefore, the design of the preform, its shape and volume, are of paramount importance. This article describes work which was carried out to develop the design of the preform’s shape and size subsequently used in forging trials of the proposed new forging technology. The considered preform designs were assessed using the finite element method analysis and experimental testing.

## 2. Research Methodology

### 2.1. Premise of the New Technology and Material

**Figure 1:**
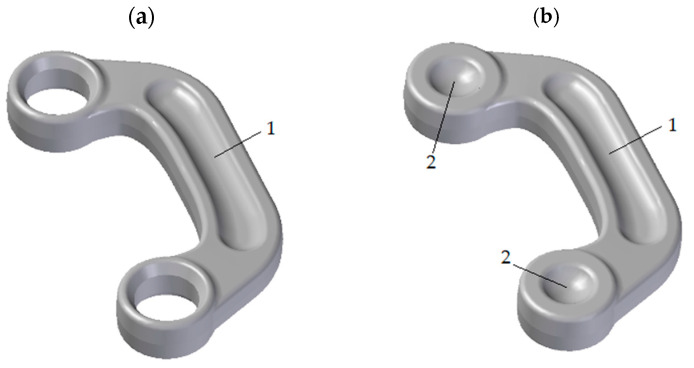
3D model of the final connector part (**a**) and the developed connector forging (**b**). 1—groves in head connecting section; 2—recesses connector’s head.

The connector part shown in Figure 1a was selected in order to assess the potential of the new screw forging process from cast preforms. High-strength ZK60 magnesium alloy, which is in the group of Mg-Zn-Zr alloys, was selected for testing [34,35,36]. The chemical composition of the ZK60 cast magnesium alloy used in this research is shown in Table 1.

Based on the geometrical model of the connector part (Figure 1a), a forging was designed, whose 3D model is shown in Figure 1b.

It was assumed that in the new technology of high-strength Mg alloy forging, the cast preforms geometry will be optimized relative to the shape of the forging of the selected connector part. This was possible based upon computer simulations of the process of metal forming of the preforms, which allowed their multi-variate analysis. The use of a cast preform whose shape closely reflects the outline of the forging in the die parting plane has a favorable impact on the metal flow kinematics, particularly during the deformation of less ductile grades of Mg alloys, which allows for a correct, defect-free product of a more accurate shape and dimensions to be obtained. Reducing the number of operations needed to produce the forging allows for better material and energy savings. It is assumed that in the new method of manufacturing parts from high-strength Mg alloys, the process will be carried out by means of die-forging from cast preforms in a single operation in a finishing impression applying typical forging machines (screw press) and with the application of inexpensive methods of tool heating (furnace, gas burner).

**Figure 2:**
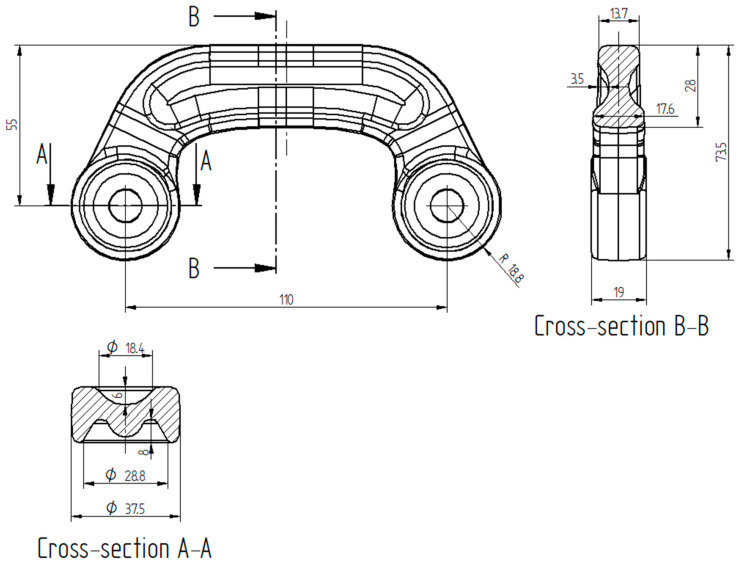
Characteristic dimensions of the connector forging in millimeters, which were altered to develop the preform.

**Figure 3:**
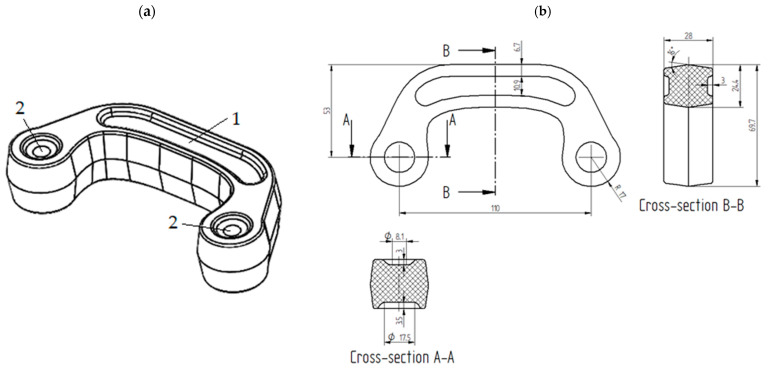
The geometry of preform #1 in millimeters: (**a**) 3D model; (**b**) characteristic dimensions: 1—groves in head connecting section; 2—recesses connector’s head.

The first considered preform was designed by modifying the shape of the forging; the height was increased from 27 mm to 28 mm, and the preform’s width and the diameter of heads were decreased; certain geometrical features were altered, e.g., 3 mm deep groves were added on both sides of the head-connecting section; some other dimensions of recesses in the connector’s heads were changed; all these are shown in sections A-A and B-B in Figure 2. The volume of the preform should be sufficient to fill the die cavity, produce strain enabling recrystallization, and create a flash with satisfactory width. Characteristic, altered dimensions of the forging are shown in Figure 2. The 3D model of preform #1 and its characteristic dimensions are shown in Figure 3a,b, respectively. The volume of this preform was 117,242 mm^3^. In comparison to the cylindrical billet used in the original three-stage process, the volume of preform #1 would allow 5852 mm^3^ (4.75%) of the ZK60 alloy per one forging to be conserved.

**Figure 4:**
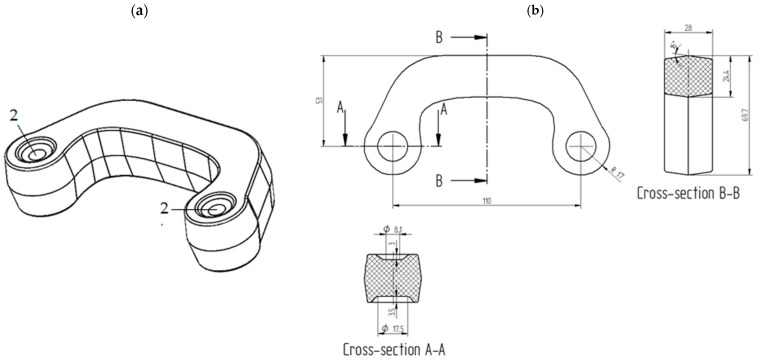
The geometry of preform #2 in millimeters: (**a**) 3D model; (**b**) characteristic dimensions: 2—recesses connector’s head.

The second considered preform was developed by modifying the shape of preform #1. The modification consisted in removing the groves from the bottom and top surfaces of the head-connecting section. This increased the volume of the preform to 123,684 mm^3^. Removing the grooves made the volume of preform #2 larger by 0.48% than the volume of the cylindrical billet used in the original three-stage forging process. The 3D model of preform #2 and its characteristic dimensions are shown in Figure 4a,b, respectively.

### 2.2. Numerical Simulation Conditions

**Figure 5:**
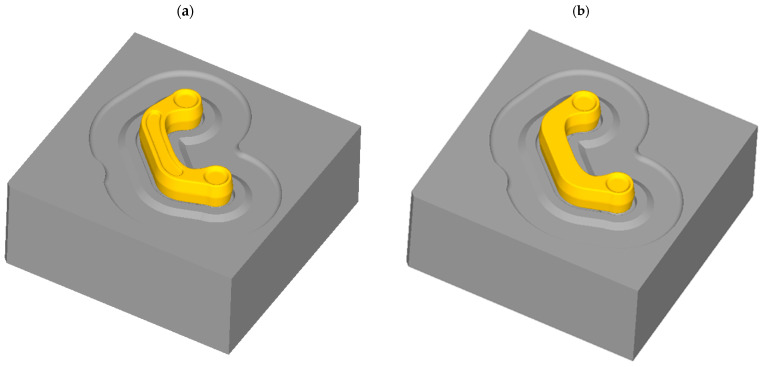
FEM model of the connector forging made from preform by variant: (**a**) #1, (**b**) #2 (top dies have been eliminated to make it easier to visualize the process).

**Figure 6:**
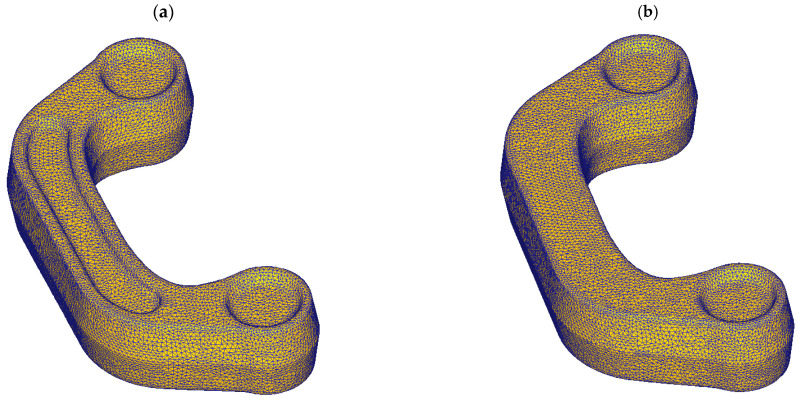
Meshed model of the connector preform by variant: (**a**) #1, (**b**) #2.

The finite element method analysis of the screw press forging of the connector using the cast preform was used to assess if the proposed preform enables a defect-free part to be forged, the distribution of strain and temperature to be computed, locations at the risk of cracking within the forged component to be indicated, and the amount of energy required for the forging process to be established. A 3D FEM model of the process (Figure 5) was created in DEFORM. Computer simulations of the investigated processes were developed on the assumption of spatial deformation and the use of a full thermomechanical model. The preforms and dies were drawn in the Solid Edge software and then imported into the DEFORM preprocessor module. The billets in the form of variant #1 and variant #2 preforms were modelled using 150,000 tetragonal finite elements (Figure 6). The simulations used the material model of the ZK60 magnesium alloy cast into sand molds developed during experimental tests. The flow stress curves used in the model were derived from compression tests carried out at three temperatures of 350 °C, 400 °C, and 450 °C, and at four strain rates of 0.01 s^−1^, 0.1 s^−1^, 1.0 s^−1^, and 10.0 s^−1^. The simulations were carried out for the preform at the temperature of 400 °C, and the temperature of both dies equals 250 °C [37,38,39]. The movement in the DEFORM software for the screw press was defined according to the machine parameters, i.e., screw press, impact energy—40 kJ, moment of inertia of moving elements—557.8 kgm^2^, and thread lead—188 mm was given. Friction on the specimen-die interface was modelled using a shear friction model with the shear factor m = 0.25 [40]. The heat transfer coefficient between the deformed metal and the tool was set to 4.5 kW/m^2^K, while that between the material and the environment equaled 0.02 kW/m^2^K [41].

### 2.3. Experimental Forging Tests Conditions

**Figure 7:**
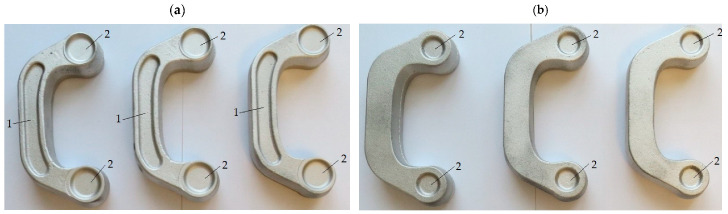
Sand casting of connector preforms according to variant: (**a**) #1, (**b**) #2. 1—groves in head connecting section; 2—recesses connector’s head.

**Figure 8:**
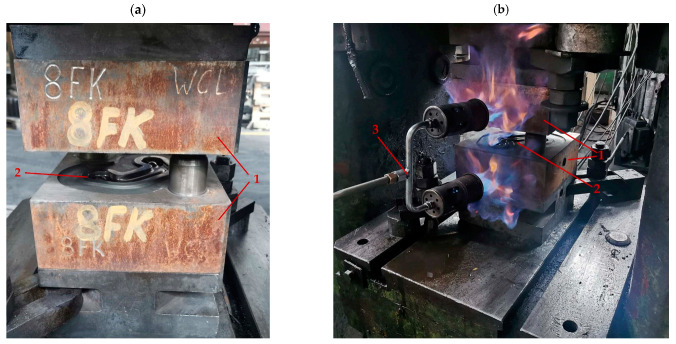
Dies with finishing impressions for the forging of connector forgings (**a**) and dies heated with a gas burner (**b**). 1—dies; 2—finishing impression; 3—gas burner.

The numerical results were verified by experimental tests performed in the same conditions as those applied in the computer simulations. Before the forging process, all preform castings (Figure 7) were subjected to two-stage homogenization at 350 °C for 10 h, and then at 450 °C for 6 h [42,43]. Forging tests of connector forgings from two preform casting geometries were performed on a screw press with the impact energy of 40 kJ and the maximum nominal pressure of 642 tons and a speed of 0.6 m/s. Before forging on the press, the dies (Figure 8a) were furnace heated to 250 °C. The temperature was maintained with gas burners during testing (Figure 8b). The preforms were heated to the forging temperature of 400 °C in an electric furnace for 40 min. After obtaining appropriate thermal conditions, both variants of preforms were placed in the finishing impression of the dies and subjected to the forging process.

After forging on the press, the correct forgings were trimmed from the flash using a saw and etched in baths with parameters [44]:

- warm water, 50–70 °C;
- cold water;
- nitric acid aqueous solution HNO3 (approx. 20–35%);
- cold water;
- sodium hydroxide aqueous solution NaOH (approx. 10%) 50–70 °C [44].
  Subsequently, the following heat treatment was applied [42,43,45]:
– supersaturation—heating from ambient temperature T = 20 °C to T = 420 °C in 40 min; then from T = 420 °C to T = 430 °C in 15 min; holding at the temperature of T = 430 °C for 90 min and cooling in water;
– aging in temperature T = 175 °C for 17 h and cooling in air.

The heat treatment was carried out in a LAC resistance electric furnace equipped with a protective gas connection. During the heat treatment, argon was fed into the furnace chamber at a flow rate of 5 L/min. In addition, batches of samples were protected with a fire blanket to prevent ignition when the furnace door was opened.

### 2.4. Conditions for Studying the Structural and Mechanical Properties of Forgings

The structure and mechanical properties were studied to assess the quality of the formed preforms and forgings. Microstructural tests were conducted in the cross-section of the preforms and forgings. Samples were prepared for structural testing. They were treated with a grinding and polishing process. Pre-grinding was performed using a SiC-coated abrasive disc with a grain size of 400 for 60–120 s. Subsequently, 9 µm diamond suspension polishing was applied for 3 min. A further step was polishing using a diamond suspension with grain sizes up to 3 µm for 180–360 s. Subsequently, polishing was conducted using colloidal silica with a grain size of 0.05 µm for 240 s. The samples were thoroughly rinsed with alcohol between each step to prevent surface oxidation. After the polished surfaces were developed, the samples were etched by immersion and gentle agitation for 10–15 s in a solution of the following composition: 100 mL ethanol, 10 mL distilled water, 10 mL acetic acid, and 5 g picric acid etchant. Microstructural studies were conducted on a Nikon MA200 optical microscope. Hardness measurements were performed by the Vickers method with application of a Future-tech FM800 hardness tester. Measurements were performed on an HV scale of 0.5 in according to PN-EN ISO 6507-1: 2006.

**Figure 9:**
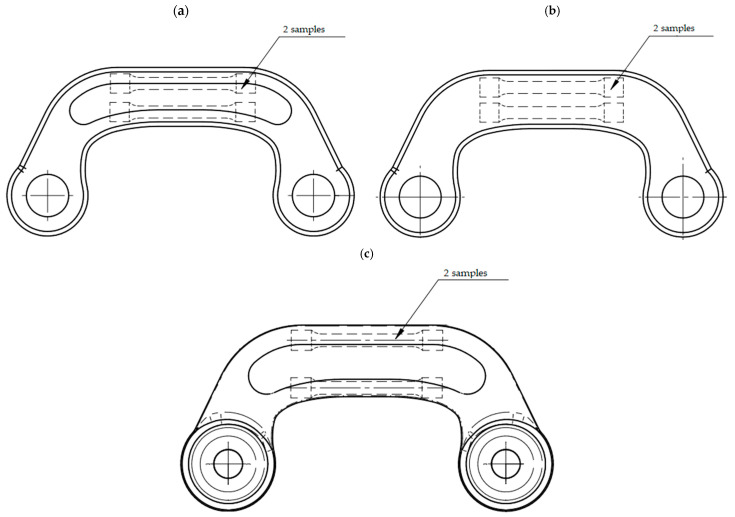
Sampling locations for testing mechanical properties: (**a**) preform #1; (**b**) preform #2; (**c**) forging.

Mechanical tests were conducted on samples made from preforms and forgings. Three iterations each were performed for testing mechanical properties. Figure 9 shows, for example, the place from where samples for tests of mechanical properties were derived from.

**Figure 10:**
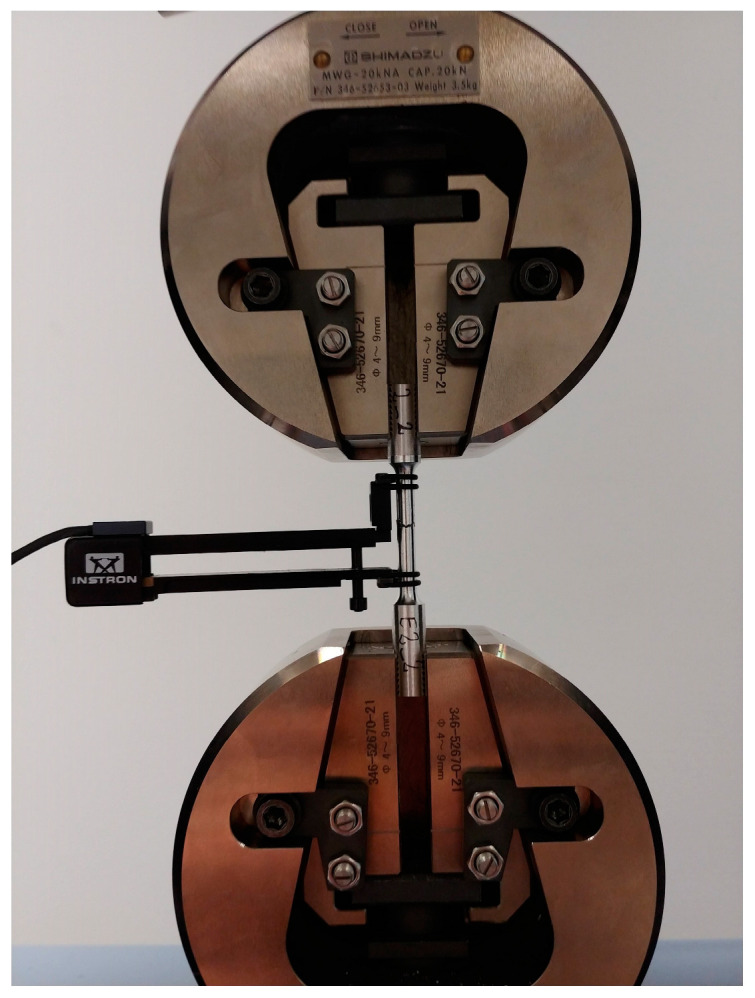
An example specimen made from a connector forging placed in a testing machine.

Sample geometry and test procedures were used in accordance with ISO 6892-1. The tensile test was carried out at the room temperature of 20 °C on a Shimadzu AG-X plus 20KN machine (Shimadzu Corporation, Kyoto, Japan) and equipped with a longitudinal extensometer to measure deformations and to control the test speed (Figure 10). The test speed was variable. For the elastic range, strain was controlled by the signal from the extensometer, and the speed was 0.025%/s. The traverse displacement speed in the plastic flow range was 0.9 mm/min.

## 3. Results and Discussion

### 3.1. Results of Numerical Simulations

**Figure 11:**
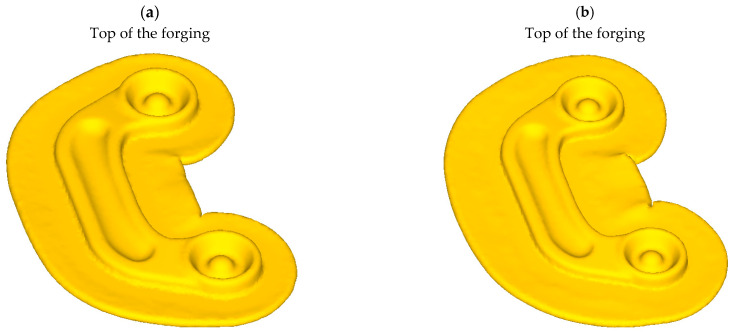

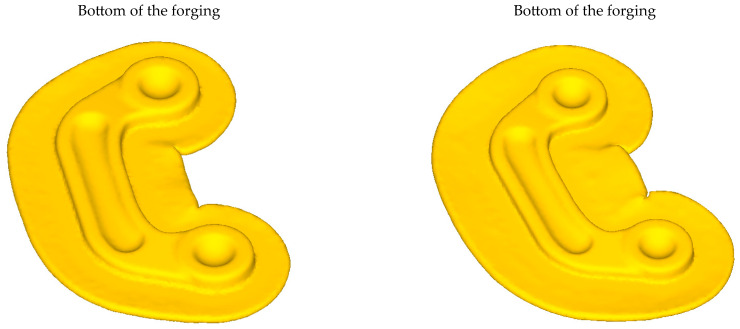
The shape of the connector forged using preform variant: (**a**) #1, (**b**) #2.

The numerical results demonstrate that the ZK60 magnesium alloy connector can be produced by the proposed screw press forging method from the cast preform. Figure 11 shows the FEM simulation of the shape of the finished part with visible flash. The numerical results also provided information on the distribution of deformation, temperature, and damage in the connector forgings [44,46,47].

**Figure 12:**
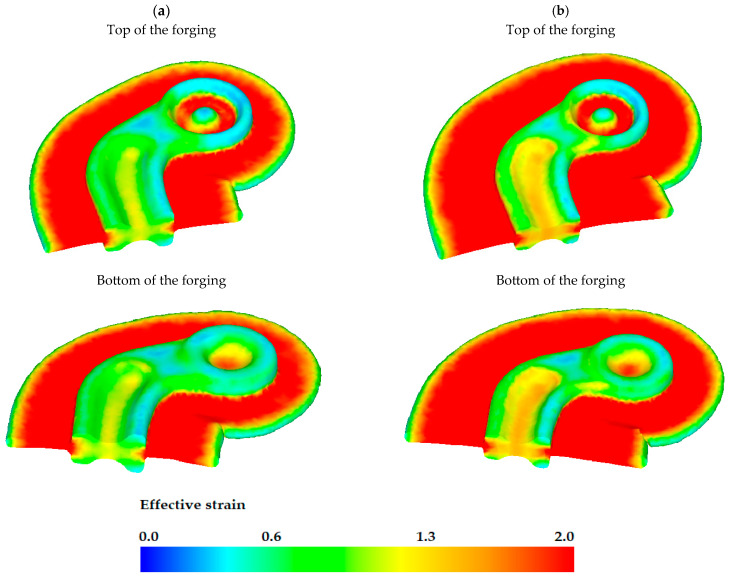
Effective strain in formed forging in its cross-section obtained from preform variant: (**a**) #1, (**b**) #2.

The distribution of the effective strain in the forging upon completing the final forging operation is illustrated in Figure 12. It can be observed that the distribution of the effective strain in the forgings is not uniform. The highest strains occur in the grooves located in the heads of the connector forgings. The highest strains also occur in the flash, which is typical of die forging processes.

**Figure 13:**
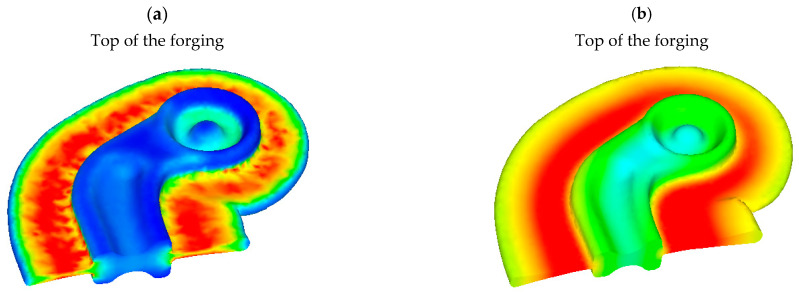

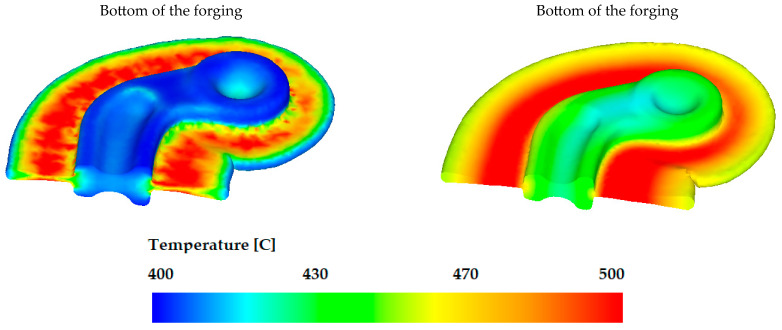
Distribution of temperature in the formed forging in its cross-section obtained from preform variant: (**a**) #1, (**b**) #2.

The temperature fields at the end of the forging process conducted on the screw press are shown in Figure 13. It can be noticed that the highest temperature in the formed forgings is 500 °C, which occurs in the area of the flash. In other areas, temperatures do not exceed 460 °C.

With strain and temperature analysis, the study investigated the risk of crack occurrence in the forgings. The modified form of the Cockroft–Latham (C-L) failure criterion was input into the theoretical analysis of this phenomenon in the DEFORM software [44,47]. The software defines the areas at risk of cracking on the basis of this criterion described by the following formula:

(1) $$\int_{0}^{\varepsilon_{p}}\frac{\sigma_{\max}}{\sigma_{H}}d\varepsilon =C_{1}$$

where: *σ_H_*—equivalent stress according to Huber’s hypothesis, *σ*_max_—maximum principal stress, ε—strain intensity, C_1_—integral value.

The Cockroft–Latham criterion establishes that when the work performed by tensile stresses in uniform tension reaches a certain critical value C_1_ = CCL, plastic fracture of the material occurs.

**Figure 14:**
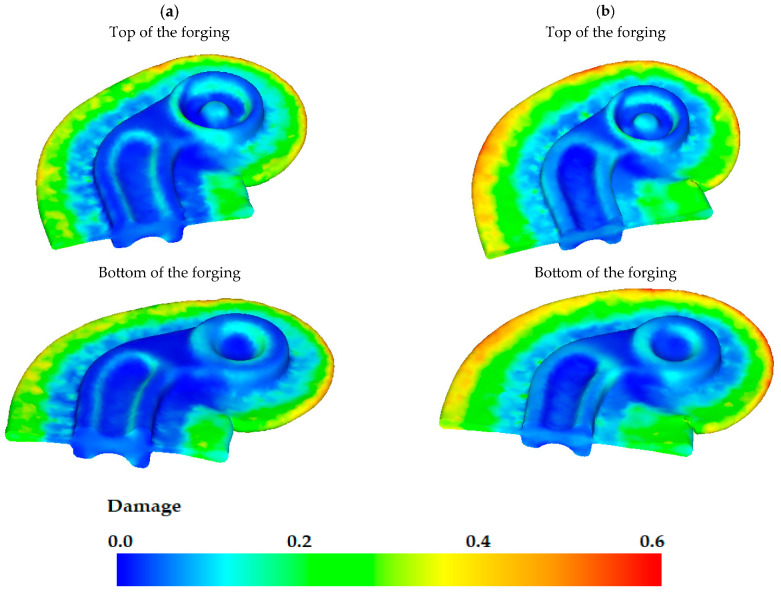
C-L damage criterion in formed forgings in its cross-section obtained from preform variant: (**a**) #1, (**b**) #2.

Example C-L damage criterion distributions in forgings after the screw press forging process are illustrated in Figure 14. The findings reveal that C-L damage criterion values are highest in the flash.

FEM analysis enabled the establishment of the amount of energy required to complete the forging process utilizing the two designed preforms. The impact energy consumed for preform #1 was 31.4 kJ. More energy was used for preform #2. The forging process had to be executed in two blows. With the first blow, the energy was 31.3 kJ, and the second blow was 16 kJ. Therefore, the forging process for preform #2 is more energy-intensive and less efficient.

### 3.2. Results of Experimental Forging Investigations

**Figure 15:**
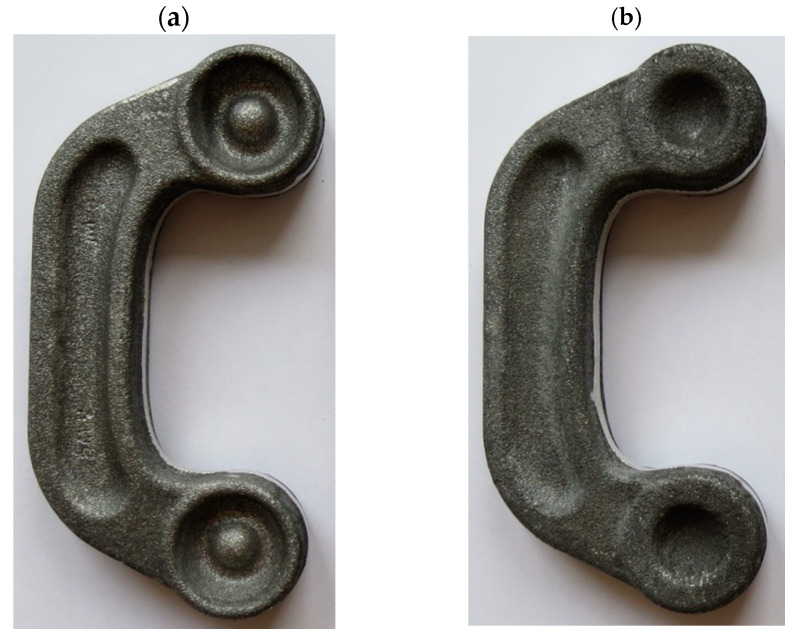
Magnesium alloy ZK60 connector forgings formed in the screw press forging process from preform #1 after trimming the flash: (**a**) top view, (**b**) bottom view.

**Figure 16:**
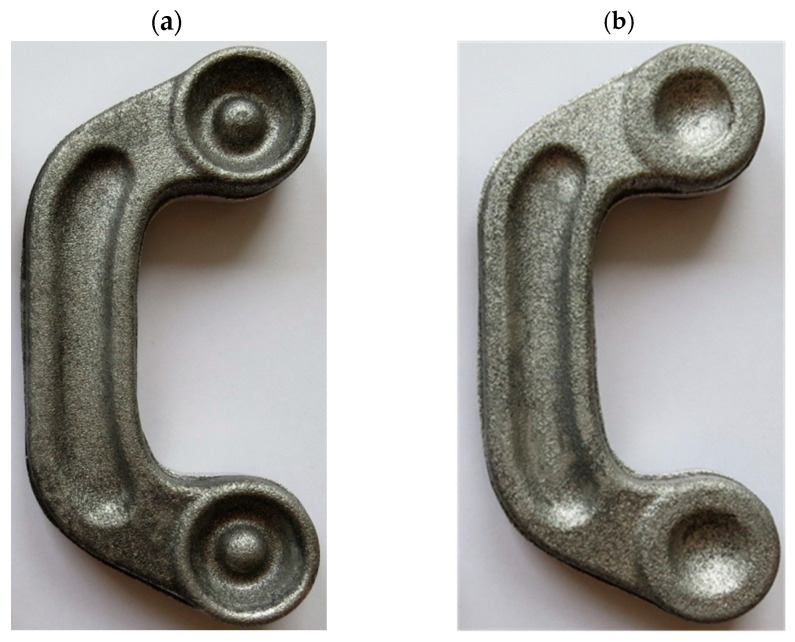
Magnesium alloy ZK60 connector forgings formed in the screw press forging process from preform #2 after trimming the flash: (**a**) top view, (**b**) bottom view.

The experimental results confirm that ZK60 magnesium alloy connectors can be produced by the proposed screw press forging method from the cast preform. Following surface cleaning and visual inspection, the produced forgings were found to be defect-free for both designed preforms. Figure 15 and Figure 16 show the forgings after flash trimming.

### 3.3. Results of Structural and Mechanical Properties Analysis of Forgings

**Figure 17:**
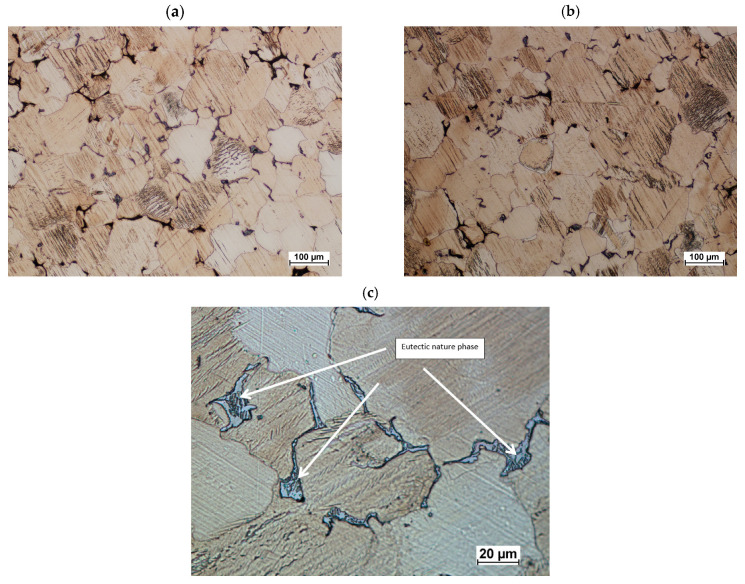
Cast of a connector preform made from ZK60 magnesium alloy according to variant: #1 (**a**,**c**) and #2 (**b**).

Based on microstructural studies of ZK60 alloy cast connector preforms, the structures shown in Figure 17 were achieved. The raw casting is characterized by a typical microstructure for this alloy. Grains of varying sizes and shapes are visible. The grains, on the entire cross-section, are relatively homogeneous in terms of their geometry. The average grain diameter for the tested castings was 63–65 μm. Detailed observations have shown that the second-phase precipitates have, at least in part, a morphology typical of eutectic precipitates (Figure 17c).

**Figure 18:**
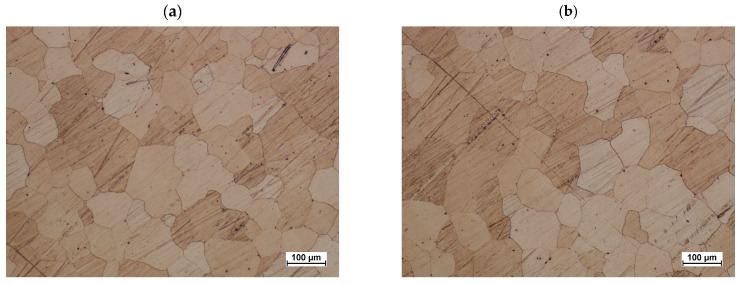
Cast of a connector preform made from ZK60 magnesium alloy after homogenization according to variant: (**a**) #1, (**b**) #2.

The structure after homogenization is fully homogenized (Figure 18). Equiaxial grains of different sizes are present in the structure. Distinct grain boundaries consist mostly of interconnected rectilinear sections. The disappearance of eutectic separations testifies to the regularity of the parameters of the homogenization process. No significant grain growth was observed. The average grain diameter does not exceed 70 µm.

**Figure 19:**
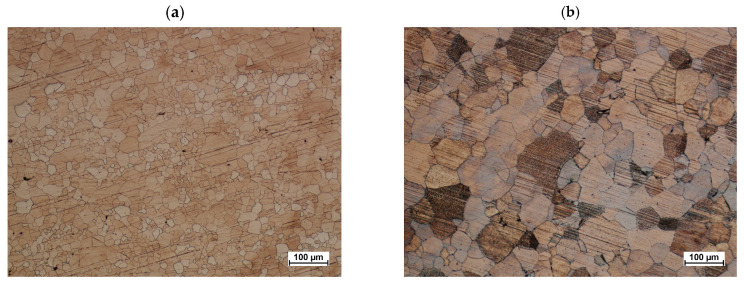
Connector forging forged from preform variant #1 (**a**) and after heat treatment (**b**).

The microstructure after forging on a screw press from preform #1 is shown in Figure 19a. The grains are mostly regular and covered by the process of dynamic recrystallization (DRX). There are few single large grains deviating in size from the remaining ones in the microstructure of the central part. This is a typical mechanism observed for many magnesium alloys where, as a result of twin boundary nucleation, fine recrystallized grains with non-basal orientation are formed, especially in the flow bands, and such a structure is referred to as bimodal [48]. A relatively high temperature of deformation has a positive effect on the dynamics of dynamic recrystallization, therefore its final stage can be observed in the microstructure, where a significant amount of grains is recrystallized, and single, larger grains are no longer elongated in the direction of deformation.

After supersaturation and artificial aging, the microstructure recrystallized throughout the studied area (Figure 19b). Regular grains with a similar size distribution dominate throughout the studied cross-section, and their average diameter was 42 µm.

**Figure 20:**
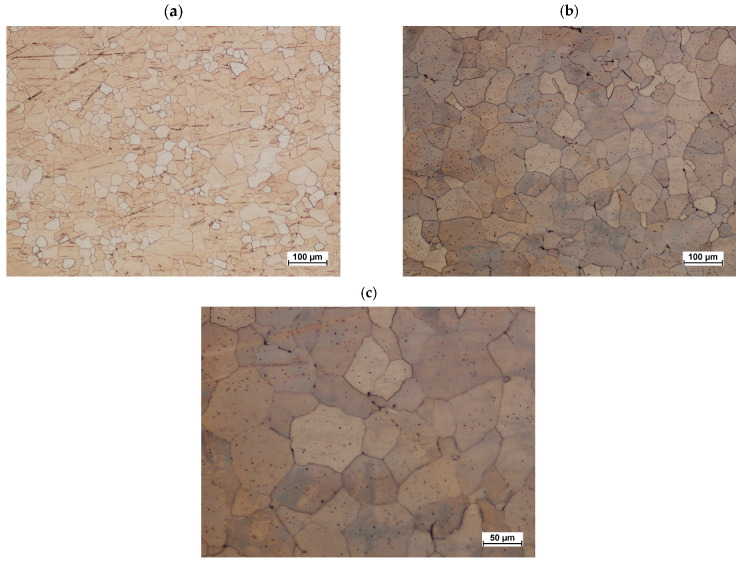
Connector forging forged from preform variant #2 (**a**) and after heat treatment (**b**,**c**).

**Figure 21:**
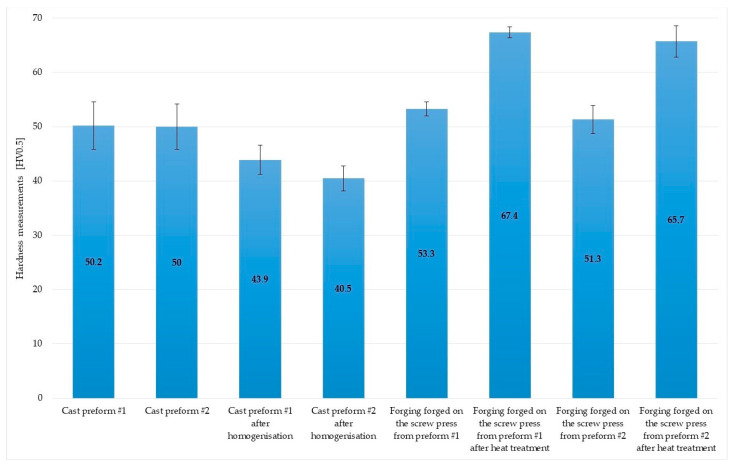
Results of hardness measurements of the connector preforms and forgings made from ZK60 cast alloy.

The post-forging variant #2 microstructure is presented in Figure 20a. Grains across all areas are regular. The microstructure does not exhibit streak formation. This proves the emergence of the dynamic recrystallization across the studied area. The microstructure of heat-treated samples produced on the screw press is presented in Figure 20b. Grains are regular. Their sizes across the studied cross-section do not differ significantly, and their average diameter is 44 µm. A characteristic coloring of grains during the etching process confirms the emergence of secondary separations and indicates the alloy’s hardening. Figure 21 shows the hardness measurement results for the connector’s preforms and forgings made from ZK60 cast magnesium alloy.

Hardness of the unprocessed alloy is 50–50.2 HV on average. After homogenization, hardness dropped to 40.5–43 HV. Post-forging hardness grows in relation to that in the post-homogenization stage. Hardness of samples produced on the screw press from preform #2 and #1 is relatively close to the average (51.3 and 53.3 HV). Hardness of all samples after full heat treatment increases considerably up to 65.7 and 67.4 HV.

**Figure 22:**
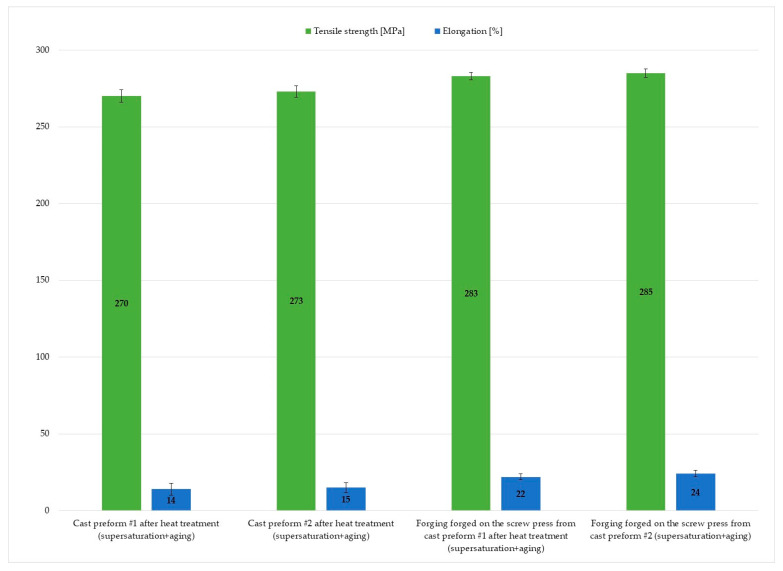
Static tensile test results of specimens made from connector’s preforms and forgings from ZK60 cast alloy.

Figure 22 presents the results of mechanical properties tests of connector forgings formed on a screw press. The results indicate that the forging process increases ductility when compared with the ductility of cast materials. The cast material reached 14% of elongation for preform #1 and 15% for preform #2. In comparison, for the forged alloy, the values reached 22% for preform #1 and 24% for preform #2. The ultimate tensile strength values for forgings forged from the two preforms were similar and equaled 270 MPa and 273 MPa.

The increase in strength and formability of the alloy after the process is associated with several mechanisms. On the one hand, the precipitation strengthening causes that the dislocation movement is blocked by evenly distributed fine precipitates of the second phase. On the other hand, the homogenization of the alloy eliminates the structural effects resulting from the presence of large precipitates on the grain boundaries with a hard but short character, which at the same time cause stress concentration and susceptibility for brittle fracture. Another factor that may be important in this case is the Hall–Patch effect described in more detail in the literature [49].

## 4. Summary and Conclusions

The aim of this study was to introduce an innovative method for producing ZK60 magnesium alloy connector forgings from cast preforms by screw press forging. The numerical and experimental results demonstrate that the proposed screw press forging process is a viable way of producing forgings of the connector from the designed geometry of preforms with the required shape. The new method ensures a larger efficiency of the process due to the shortening of forging time limited to a single forging operation, as well as considerable material savings compared to previously applied methods that consisted in forming the connector by multistage forging or from casts by means of machining operations. In this field, further research will include the study of the principles of topological optimization for the design of the parts available in CAD and simulation software. The novel process involving the forging of cast preforms enables a higher quality of the produced connectors to be obtained in relation to those machined from castings. This higher quality obtained in the course of the new process stems from a more favorable structure due to the grain size reduction and unification via metal deformation, which offers improved tensile and functional properties. Good quality of the surface stems from the impact of tools upon the processed material. As a consequence, the porosity and irregularity of the surface frequently emerging in castings are avoided.

The results demonstrate that ZK60 cast magnesium alloys maintain good plasticity in the screw press die forging process. According to the literature, the forging of magnesium alloys is usually performed by means of hydraulic presses or mechanical presses equipped with systems for tool preheating and at low operating speeds. Magnesium alloys are very seldom subjected to screw press die forging.

The study confirms the effectiveness of numerical simulations based on the finite element method. They enable multivariate analysis to be carried out at the stage of process design, which allowed the geometry of preforms to be established for experimental tests, as well as for experimental tests to be scheduled in a more detailed manner, and their costs to be reduced. This leads to decreased production costs and a shorter time of industrial implementation of new technologies.

Magnesium alloys are one of the group of lightweight, structural materials regarded as crucial to the future of various industries. For this reason, it is important to carry out more research on the technology development of manufacturing magnesium alloy products from cast preforms using screw presses, which have a much higher capacity than hydraulic presses. Successful investigations of the forging process of ZK60 alloy forgings on a screw press demonstrate that research should continue for other ductile magnesium alloys.

## Figures and Tables

**Table 1:** Chemical composition of the ZK60 cast magnesium alloy used in the experiment (wt%; producer’s certificate, NeoCast—Light Weight Technologies).

Mg	Zn	Zr	Mn	Fe	Si	Cu	Al	Ni
balance	6.04	0.78	0.02	0.001	0.005	0.001	0.001	0.001

## Data Availability

Data are contained within the article.
